# Tribological and Mechanical Performance of 3D-Printed Polymers for Water-Lubricated Sliding Bearings

**DOI:** 10.3390/ma19173593

**Published:** 2026-08-24

**Authors:** Jolanta Krupa, Jędrzej Blaut, Mariusz Warzecha, Mateusz Kozioł, Łukasz Breńkacz

**Affiliations:** 1 Faculty of Mechanical Engineering and Robotics, AGH University of Krakow, Al. A. Mickiewicza 30, 30-059 Krakow, Poland; krupa@agh.edu.pl (J.K.); mwarzech@agh.edu.pl (M.W.); makoziol@agh.edu.pl (M.K.); lbrenkacz@imp.gda.pl (Ł.B.); 2 Institute of Fluid-Flow Machinery, Polish Academy of Sciences, Fiszera 14, 80-231 Gdansk, Poland

**Keywords:** fused filament fabrication, additive manufacturing, tribology, sliding bearings, polymer composites

## Abstract

**Highlights:**

- TPU + GF showed the lowest wear rate despite having the lowest hardness and modulus of elasticity.
- PLA suffered the most severe abrasive wear, generating flake-like debris.
- ABS demonstrated the most stable friction coefficient and superior post-test surface smoothness.
- Surface compliance in additive manufacturing polymers can be leveraged to reduce wear in sliding contact applications.
- Microindentation hardness alone is insufficient to predict wear resistance in 3D-printed polymers.
- Flexible composites like TPU + GF are promising candidates for water-lubricated bearing applications based on their wear resistance during dry startup screening.

**Abstract:**

This paper presents the results of experimental studies on the mechanical and tribological properties of samples produced using the fused filament fabrication method. These studies aim to identify a potential material for a water-lubricated sliding bearing that will be manufactured using 3D printing. Printed specimens fabricated from four different materials were analyzed for surface profile, mechanical properties via the instrumented indentation method, and tribological behavior using a pin-on-disc setup. Although increased hardness is generally associated with improved resistance to wear, the material that exhibited the lowest wear in the pin-on-disc tests was characterized by the lowest hardness among all investigated materials. The findings suggest that other aspects, including wear mechanisms, wear products, and tribolayer development, may also exert a substantial influence.

## 1. Introduction

Additive manufacturing (AM), particularly fused filament fabrication (FFF), has emerged as a transformative technology that extends beyond rapid prototyping to produce functional components with complex geometries and enhanced design freedom [1]. Among various AM techniques, FFF represents the most widely adopted method for processing thermoplastic polymers due to its accessibility, cost-effectiveness, and versatility in creating lightweight, durable components for demanding applications [2]. 

However, the current trend is to use AM not only to fabricate prototypes but also to produce end-use components. As a result, understanding the structural behavior of printed components under different loading conditions is important to accelerate the adoption of this manufacturing process.

The mechanical and tribological performance of FFF-printed parts is fundamentally influenced by process parameters, material selection, and the resulting anisotropic microstructure that develops during layer-wise deposition [3]. Commonly used engineering thermoplastics such as polylactic acid (PLA), polyethylene terephthalate glycol (PETG), acrylonitrile butadiene styrene (ABS), and thermoplastic polyurethane (TPU) offer significant advantages in terms of processability and cost-effectiveness, making them attractive candidates for functional applications [4].

Recent investigations have demonstrated that process parameters, including infill density, build orientation, layer thickness, and raster angle, significantly influence the tribological behavior of FFF-printed polymers [5]. Studies have shown that optimizing these parameters can substantially reduce the coefficient of friction and wear rates, with reports that proper parameter selection can halve material volume loss due to wear in reinforced PLA systems [4].

A recent study addressed the limited availability of data on the properties of advanced FFF-printed composite materials, particularly carbon-fiber-reinforced polymers. The authors investigated a range of commercially available filaments, including PETG Carbon, FiberFlex 40D, PETG (Sunlu), HIPS (Devil Design), TPU (F3D Filament), PA + 15CF (Rosa 3D), PETG (3DActive), and PLA (3DActive). Test specimens were fabricated using Prusa MK4 printers with 100% infill, while the printing temperatures were optimized to ensure adequate interlayer bonding and structural integrity. The materials were evaluated through tensile and three-point bending tests, scanning electron microscopy (SEM) of fracture surfaces, and cytotoxicity assessments [6].

The study conducted by Li et al. showed that the orientation of carbon fibers relative to the sliding direction has a significant influence on tribological performance. Fibers aligned parallel to the sliding direction resulted in lower wear rates and friction coefficients than those oriented normal to the sliding direction, where fiber fracture was more pronounced. The authors also highlight the importance of a specialized adhesive layer to ensure strong bonding between the printed layers and the metal substrate. By analyzing these morphological traits, the researchers developed a predictive mathematical model to estimate tribological behavior based on specific fiber patterns. Ultimately, the findings provide a framework for optimizing the design of high-performance, self-lubricating mechanical components through precise 3D printing techniques [7].

Beyond friction and wear resistance, a material’s suitability for bearing applications should also be evaluated based on the dynamic behavior of the supported rotor. Bearing stiffness and damping directly affect critical speeds, vibration modes, response amplitudes, and the stability of rotor–bearing systems. Zhou et al. showed that these dynamic support parameters can be identified from experimentally measured rotor unbalance responses. Although their study focused on active magnetic bearings, it underscores the broader importance of reliable stiffness and damping characterization in rotating machinery. In polymer sliding bearings, material compliance, bearing geometry, surface condition, and wear-induced dimensional changes may similarly influence the effective dynamic characteristics of the bearing support [8].

It should be emphasized that the indentation modulus, E_IT_, is a local material parameter and should not be interpreted as the equivalent stiffness of the complete bearing support. Effective bearing stiffness and damping are system-level, operating-condition-dependent quantities that are also influenced by bearing geometry, clearance, contact conditions, lubrication, assembly, and rotor flexibility. Xu et al. demonstrated that such dynamic bearing coefficients can be identified under rotating conditions from experimentally measured rotor unbalance responses. Although their investigation focused on active magnetic bearings, the distinction between local material properties and system-level support parameters remains applicable to other bearing concepts. Consequently, the present H_IT_ and E_IT_ values should be regarded primarily as material-screening indicators for interpreting friction and wear, while component-level rotor-bearing experiments will be required to determine the effective stiffness and damping of a complete 3D-printed sliding bearing [9].

Despite these advances, the relationship between mechanical properties and tribological performance remains insufficiently understood. While M. Norani et al. [2] established that the coefficient of friction and wear rates are relatively dependent on the elastic modulus in ABS components, comprehensive analyses linking nano-scale mechanical properties with macroscopic friction behavior under application-relevant conditions are notably absent from the current literature [2]. This knowledge gap is particularly critical in the context of sliding systems operating under environmentally sustainable lubrication regimes, such as water-lubricated sliding bearings, where material compliance, load-carrying capacity, and the ability to support stable lubricating films are key performance factors.

Therefore, this study aims to establish quantitative correlations between microindentation-derived mechanical properties, specifically modulus of elasticity (E_IT_) and hardness (H_IT_), and the tribological response of FFF-printed polymers. It is hypothesized that these properties govern the deformation behavior and real contact area during sliding, thereby significantly influencing frictional performance. In particular, materials exhibiting lower stiffness and hardness are expected to demonstrate increased compliance, which may reduce localized stress concentrations and wear during severe contact regimes, such as startup or temporary disruption of the water film. The outcomes of this work provide an initial material-screening framework for polymer-based components intended for water-lubricated bearing applications. In contrast, stiffer materials may provide improved load-bearing capacity at the expense of higher friction. The outcomes of this work are intended to support material selection strategies for polymer-based components in water-lubricated bearing applications and contribute to the development of more efficient and environmentally friendly tribological systems.

## 2. Materials and Methods

### 2.1. Sample Preparation

The investigated specimens were manufactured using the fused filament fabrication (FFF) process from four commercially available materials: PLA—Polylactic Acid (Devil Design, Mikołów, Poland), PETG—Polyethylene Terephthalate Glycol (Prusa Research, Prague, Czech Republik), ABS—Acrylonitrile Butadiene Styrene (Fiberlogy, Brzezie, Poland), and TPU + GF—glass-fiber-reinforced thermoplastic polyurethane (ROSA 3D Filaments, Hipolitów, Poland). All specimens were prepared using Bambu Lab P2S 3D printer (Shenzhen, China), the same slicing software, identical printing strategy, and a single FFF printer to ensure repeatable manufacturing conditions. The selected materials are compatible with mid-range enclosed FFF printers and can be processed without specialized equipment. Prior to printing, all filaments were dried according to the manufacturers’ recommendations, which was particularly important for the hygroscopic TPU + GF filament. A 0.4 mm nozzle and a layer height of 0.2 mm were used for all specimens. Each specimen was printed in a flat orientation (0° relative to the build plate) using two perimeter walls and 100% rectilinear infill. Previous studies have shown that the build orientation of FFF-printed polymers may influence their tribological performance due to the interaction between the layer arrangement and the sliding direction [10]. A 100% infill was selected to eliminate the influence of the designed internal infill structure on the measured mechanical and tribological properties; however, this does not preclude the presence of process-induced porosity, such as interlayer voids or local lack of fusion. The printing parameters adopted for each material are summarized in Table 1. The reported printing speed ranges correspond to the different speeds assigned to individual printing features according to the applied Bambu Studio slicing strategy. The lower values correspond to the speeds used for perimeter walls, while the higher values represent the infill printing speeds. The chamber temperature values correspond to the controlled set temperatures maintained during the printing process. Processing parameters (Table 1) were tailored to the thermal and rheological requirements of each polymer to ensure optimal layer bonding and defect-free specimens, with reduced speeds for TPU + GF to prevent feeding instability.

### 2.2. Mechanical Properties

Hardness and modulus of elasticity are important parameters for evaluating polymeric materials intended for sliding-bearing applications because they characterize the local response of the bearing surface to concentrated contact loading [11]. Hardness and modulus of elasticity are important parameters for evaluating polymeric materials intended for sliding-bearing applications because they characterize the local response of the bearing surface to concentrated contact loading [11,12].

Instrumented indentation hardness, H_IT_, describes the resistance of the material to irreversible local deformation and penetration by surface asperities or abrasive particles. A higher hardness generally improves the ability of the surface to resist indentation, plowing, and plastic deformation, although hardness alone does not determine the overall wear resistance. The modulus of elasticity, E_IT_, characterizes the local elastic stiffness of the material and therefore affects contact deformation, the real area of contact, load distribution, and the ability of the bearing surface to recover after loading. These properties are particularly relevant in water-lubricated sliding bearings, where direct interaction between opposing asperities may occur under boundary or mixed-lubrication conditions, for example, during start-up, low-speed operation, or temporary disruption of the lubricating film [13,14,15].

The local mechanical properties of the investigated polymers were determined by instrumented indentation using an Anton Paar Step 500 Surface Testing Platform (Anton Paar, Graz, Austria) equipped with a Micro Combi Tester (MCT^3^) module. Measurements were performed using a diamond Vickers indenter at a maximum applied load, P_max_, of 1 N. A total of 15 measurements were conducted for each specimen. The loading and unloading rates were both 2 N/min. After reaching the maximum load, the load was held constant for 15 s. The holding period was introduced to reduce the influence of indentation creep on the initial unloading response, which is particularly important for polymers because of their time-dependent viscoelastic and viscoplastic behavior [16].

**Figure 1:**
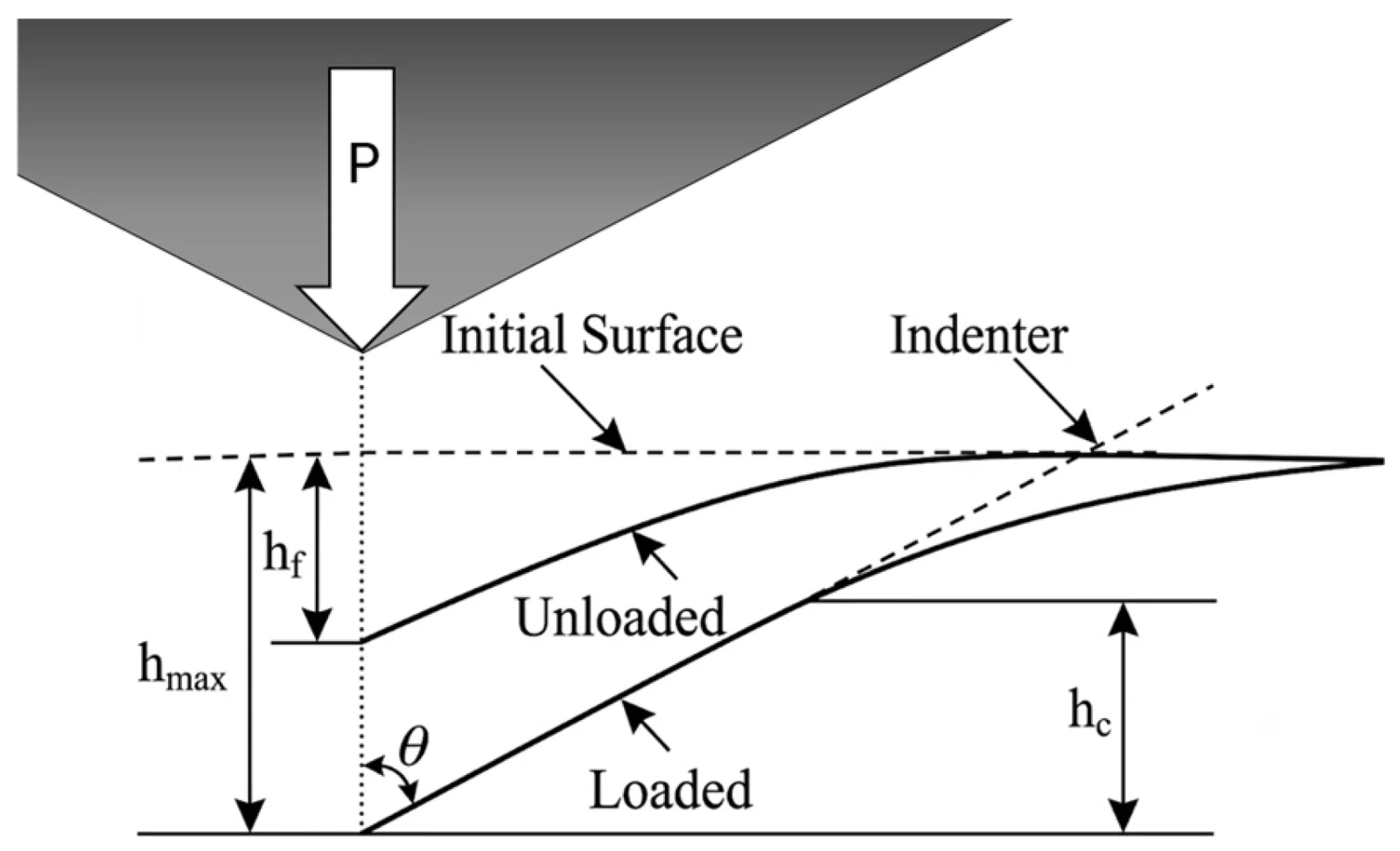
Loading and unloading during the indentation test with marked penetration and residual depths.

During each measurement schematically shown in Figure 1, the applied load, P, and the corresponding indentation depth, h, were recorded continuously. Instrumented indentation hardness, $H_{IT}$, and indentation modulus of elasticity, $E_{IT}$, were determined from the load–displacement curve using the Oliver–Pharr method [17]. The method assumes that the initial part of the unloading response is dominated primarily by elastic recovery. The following power-law relationship commonly represents the unloading curve:

(1) $$P\;\;=\;\alpha {\left(h-h_{f}\right)}^{m}$$

where α and m are fitting parameters, and $h_{f}$ is the residual indentation depth after unloading.

The contact stiffness, S, is obtained from the slope of the fitted unloading curve at the maximum indentation depth:

(2) $$S=\frac{dP}{dh}\;\;at\;\;h\;=\;h_{max}$$

where $h_{max}$ is the maximum depth of indenter during the test.

The contact depth, $h_{c}$, is subsequently calculated as:

(3) $$h_{c}=h_{max}-\varepsilon \frac{P_{max}}{S}$$

where ε is a geometrical constant associated with the indenter geometry. For a Vickers indenter, a value of approximately 0.75 is commonly employed.

The projected contact area, $A_{p}$, is then evaluated from the calibrated indenter area function at $h_{c}$. For an ideal Vickers geometry, the leading term of the area function is:

(4) $$A_{p}=25.4h_{c}^{2}$$

In practice, the calibrated area function should account for deviations from the ideal indenter geometry, including tip rounding.

The instrumented indentation hardness is determined from the maximum load and the projected contact area:

(5) $$H_{IT}=\frac{P_{max}}{A_{p}}$$

The reduced contact modulus, $E_{r}$, is calculated from the contact stiffness according to:

(6) $$E_{r}=\frac{\sqrt{\pi }}{2\beta }\frac{S}{\sqrt{A_{p}}}$$

where β is a correction factor accounting for the non-axisymmetric geometry of the indenter.

The indentation modulus of the specimen is subsequently obtained by accounting for the elastic deformation of both the investigated material and the diamond indenter:

(7) $$\frac{1}{E_{r}}=\frac{1-\nu_{s}^{2}}{E_{IT}}+\frac{1-\nu_{i}^{2}}{E_{i}}$$

where $E_{i}$ and $\nu_{i}$ are the elastic modulus and Poisson’s ratio of the diamond indenter, respectively, and $\nu_{s}$ is the Poisson’s ratio assumed for the investigated polymer, $E_{IT}$ is indentation modulus of elasticity, hereinafter referred to as modulus of elasticity.

For polymeric materials, the values obtained using the Oliver–Pharr procedure can be affected by creep during the holding and unloading stages, strain-rate sensitivity, viscoelastic recovery, surface roughness, and pile-up or sink-in around the indentation [13]. In additively manufactured polymers, additional variability may result from local porosity, interlayer boundaries, build orientation, crystallinity, and spatial variations in thermal history [13,15]. Consequently, E_IT_ should be interpreted as a local indentation modulus associated with the applied loading protocol and the investigated surface rather than as a direct replacement for the bulk tensile Young’s modulus.

The resulting H_IT_ and E_IT_ values were used to compare the resistance of the printed polymers to local irreversible deformation and their elastic response under concentrated loading. These results were subsequently employed to support the interpretation of differences in friction and wear.

### 2.3. Surface Topography and Roughness

Surface topography and roughness measurements were performed in order to characterize the surface condition of the printed pins before and after the experimental tests. The measurements were carried out using a Profilm3D (Filmetrics, San Diego, CA, USA), optical profilometer operating on the principle of white-light interferometry (WLI). This non-contact measurement technique enables the reconstruction of the surface height distribution by analyzing the interference signal generated by light reflected from the tested surface and from the reference optical path.

**Figure 2:**
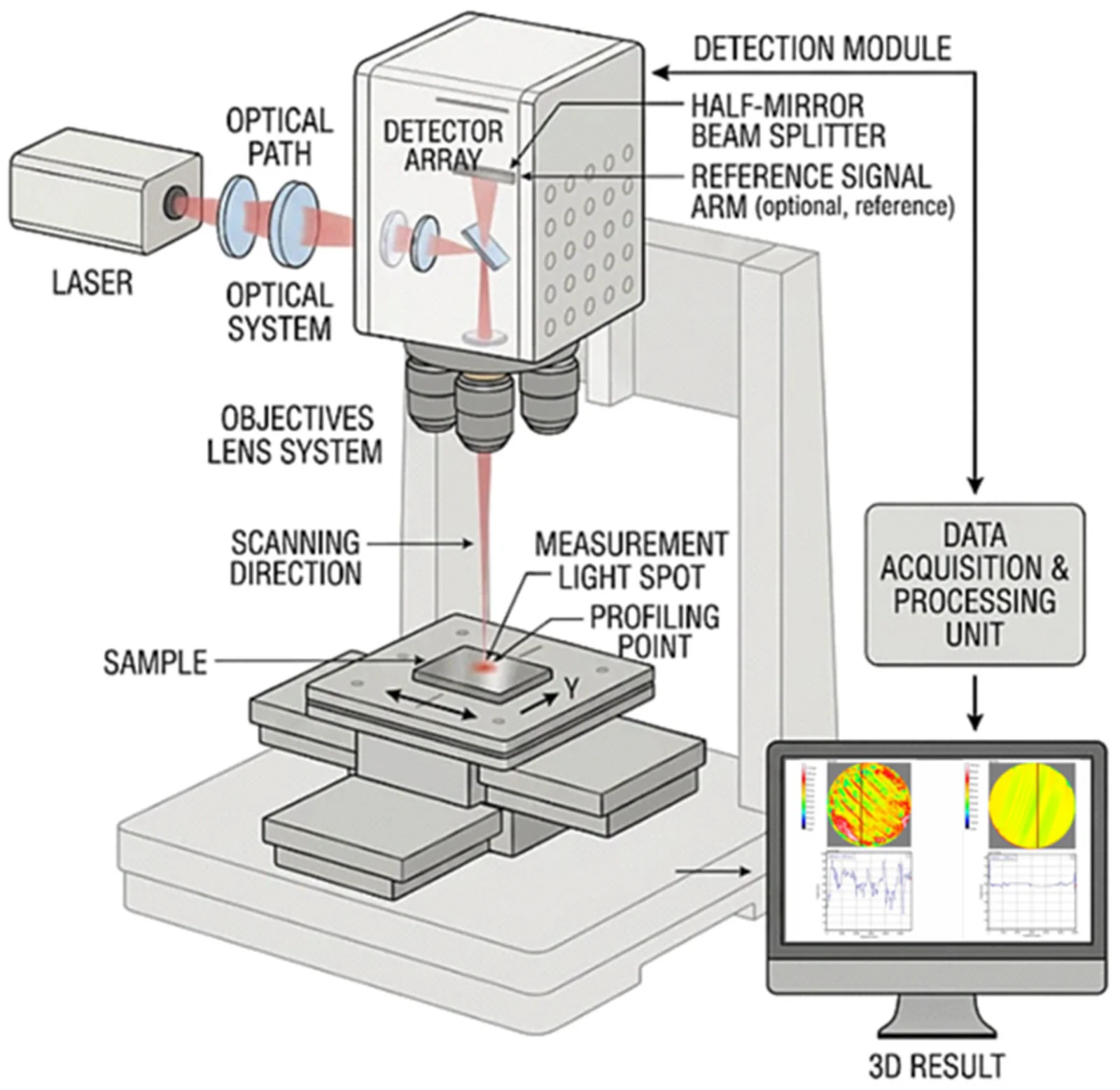
Schematic representation of the optical profilometry setup used for surface topography measurements of the printed pins. Created with assistance from Gemini 3.5 Flash and verified by the authors.

A schematic representation of the profilometric measurement setup is shown in Figure 2. During the measurement, light is directed through the optical system and the objective lens onto the surface of the specimen. The reflected signal is collected by the detection module and transferred to the data acquisition and processing unit, where the surface height map is reconstructed. As a result, three-dimensional topography maps of the measured area are obtained.

For each specimen, the measurements were performed using a 10× objective lens combined with 4× digital magnification. The digital magnification was used to improve the visualization and positioning of the measurement area, whereas the actual optical resolution was governed by the objective lens and the profilometer settings. In order to cover the full analyzed surface of each pin, 206 individual measurement fields were acquired for each specimen. This procedure made it possible to characterize the surface over the entire region of interest rather than at a single local point.

The acquired topography data were used to evaluate areal-based roughness parameter Sa (arithmetic mean height) which extends the roughness assessment to the entire measured surface. It was determined from three-dimensional surface height maps in accordance with the ISO 25178 standard [18]. In addition to Sa, the surface topography was characterized using the following parameters: maximum peak height (Sp), maximum pit height (Sv), maximum height (Sz), root mean square height (Sq), and skewness (Ssk). Since Sa is less sensitive to measurement direction and surface anisotropy, it offers a more representative description of surface texture, particularly for heterogeneous surfaces produced by additive manufacturing [19,20]. Therefore, the Sa parameter was selected as the primary roughness indicator for further analysis.

The same measurement procedure was applied to the specimens before and after the experimental test. This enabled a comparative assessment of the initial surface condition and the surface changes caused by the test. The obtained height maps and roughness parameters were therefore used to quantify the modification of the pin surface topography and to identify changes in roughness resulting from the interaction with the tested material.

### 2.4. Tribological Tests

Tribological tests were performed using a pin-on-disc configuration on T-01 Testing Machine (Łukasiewicz Research Network–Institute for Sustainable Technologies, Radom, Poland) in order to evaluate the sliding behavior of the tested material under dry friction conditions. This test method is commonly used for the comparative assessment of friction and wear of material pairs subjected to controlled sliding contact. In the applied configuration, the pin specimen made of the tested material was pressed against a rotating steel disc, producing a circular wear track on the counterface.

**Figure 3:**
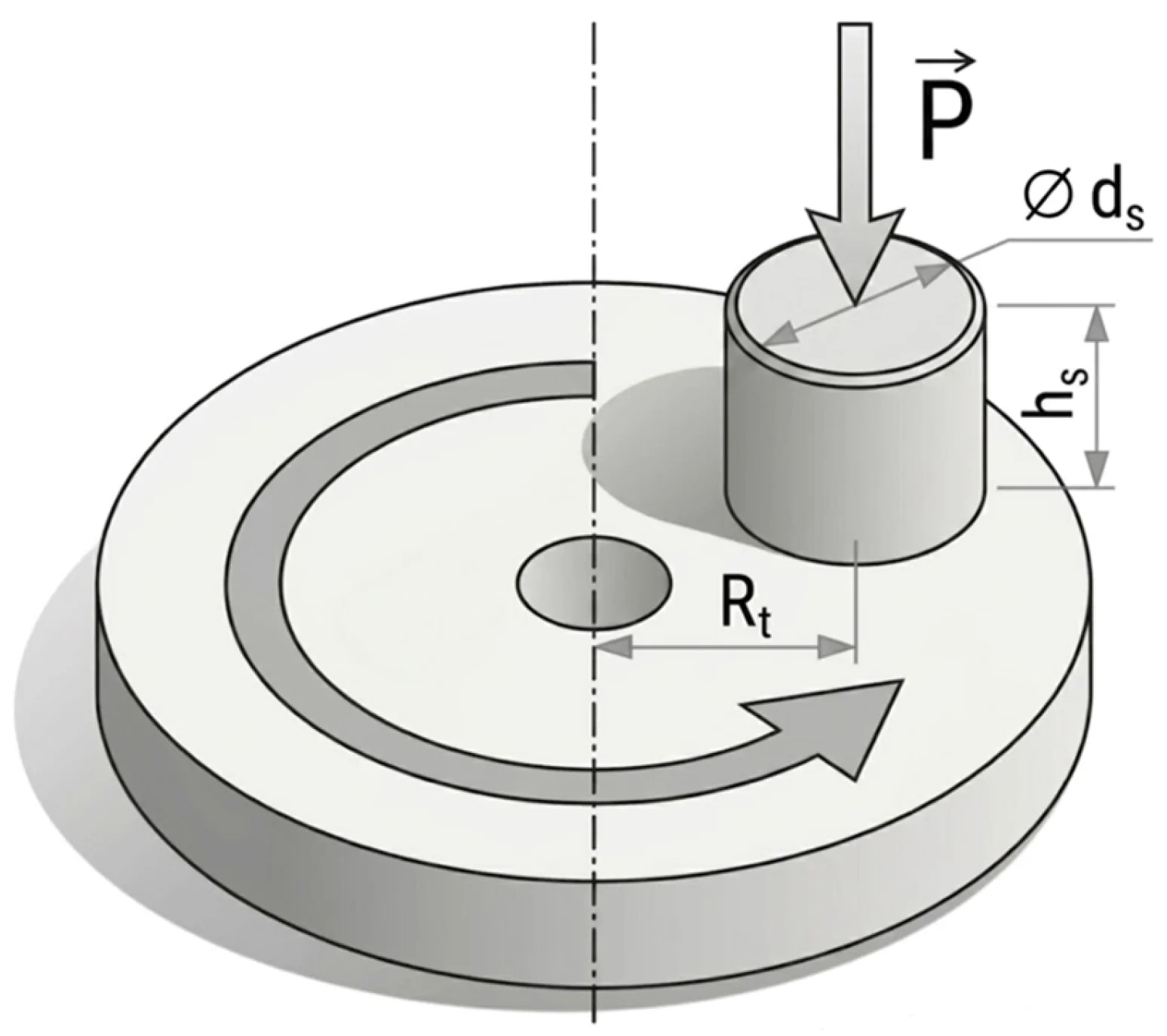
Schematic representation of the pin-on-disc test configuration: P—normal load; R_t_—friction radius; d_s_—pin diameter; h_s_—pin height. Created with assistance from Gemini 3.5 Flash and verified by the authors.

A schematic representation of the pin-on-disc test configuration is shown in Figure 3. The pin was loaded in the normal direction by a constant force P and remained in contact with the rotating disc during the test. The sliding motion was generated by the rotation of the disc, while the position of the pin with respect to the disc axis defined the friction radius R_t_. As a result, the contact point followed a circular path with a constant nominal sliding velocity. The tested pins had a cylindrical geometry, with a diameter of d_s_ = 6 mm and a height of h_s_ = 12 mm.

The counterface was a disc made of C45 steel, heat-treated to a hardness of 60 HRC. The initial surface roughness of the disc was Ra = 0.16 µm. The use of a hardened steel disc with a controlled surface finish provided repeatable counterface conditions for all tested pins. The pin specimens were manufactured from the investigated materials, and their surface condition was analyzed before and after the tribological test using optical profilometry, as described in the previous section.

The tests were carried out under dry sliding conditions, without the use of any lubricant. The normal load applied to the pin was P = 14 N. Taking into account the pin diameter d_s_ = 6 mm, this corresponded to a nominal contact pressure of approximately 0.5 MPa, calculated as the ratio of the normal load to the nominal circular contact area of the pin. The friction radius was set to R_t_ = 51.5 mm. The disc rotational speed was 186 rpm, which resulted in a linear sliding velocity of approximately 1 m/s at the contact point. For each specimen, 5 tests were carried out.

The adopted test parameters are summarized in Table 2. The selected load, sliding speed, and dry contact conditions were kept constant for all specimens, which enabled a direct comparison of the tribological response of the tested material. After testing, the pins were subjected to surface topography measurements in order to determine the changes in surface roughness and to assess the wear-related modification of the contact surface.

Dry-sliding conditions were chosen as a method for selecting materials prior to future testing under operating conditions for water-lubricated bearings. This approach is important because the greatest risk of operational wear on the bearing surface occurs during startup, when the lubricating film has not yet formed. Furthermore, standardized dry-sliding tests allow for a direct comparison of different materials and provide reproducible data, which is currently lacking in the selection of FFF-printed polymers intended for use in water-lubricated bearings. Under fully developed hydrodynamic fluid lubrication, solid contact is minimized, leading to negligible wear. Thus, dry screening represents a critical worst-case evaluation for material survival during lubricant failure or startup phase.

Prior to and after the test, each specimen was weighed using AS 82/220.R2 PLUS balance (Radwag, Radom, Poland) with an accuracy of 0.1 mg, and its height was measured using a micrometer.

In order to provide a quantitative assessment of the relationships between the investigated material properties and their tribological behavior, Pearson correlation coefficients were determined. The analysis included microindentation parameters (H_IT_ and E_IT_), surface roughness parameters measured before and after the tribological tests, and wear-related parameters expressed as mass loss and pin height change. The obtained correlations were used to identify the most significant factors influencing the wear response of the investigated materials.

## 3. Results

### 3.1. Initial Surface Profile Measurement

Surface roughness measurements were performed using a profilometer over the entire specimen surface. The results are presented in Table 3. The highest arithmetic mean height (Sa) was recorded for the PLA specimen, reaching 16.72 µm. Similar values were observed for ABS and PETG, amounting to 8.42 µm and 7.96 µm, respectively, whereas the Sa parameter for TPU + GF was equal to 11.60 µm.

**Figure 4:**
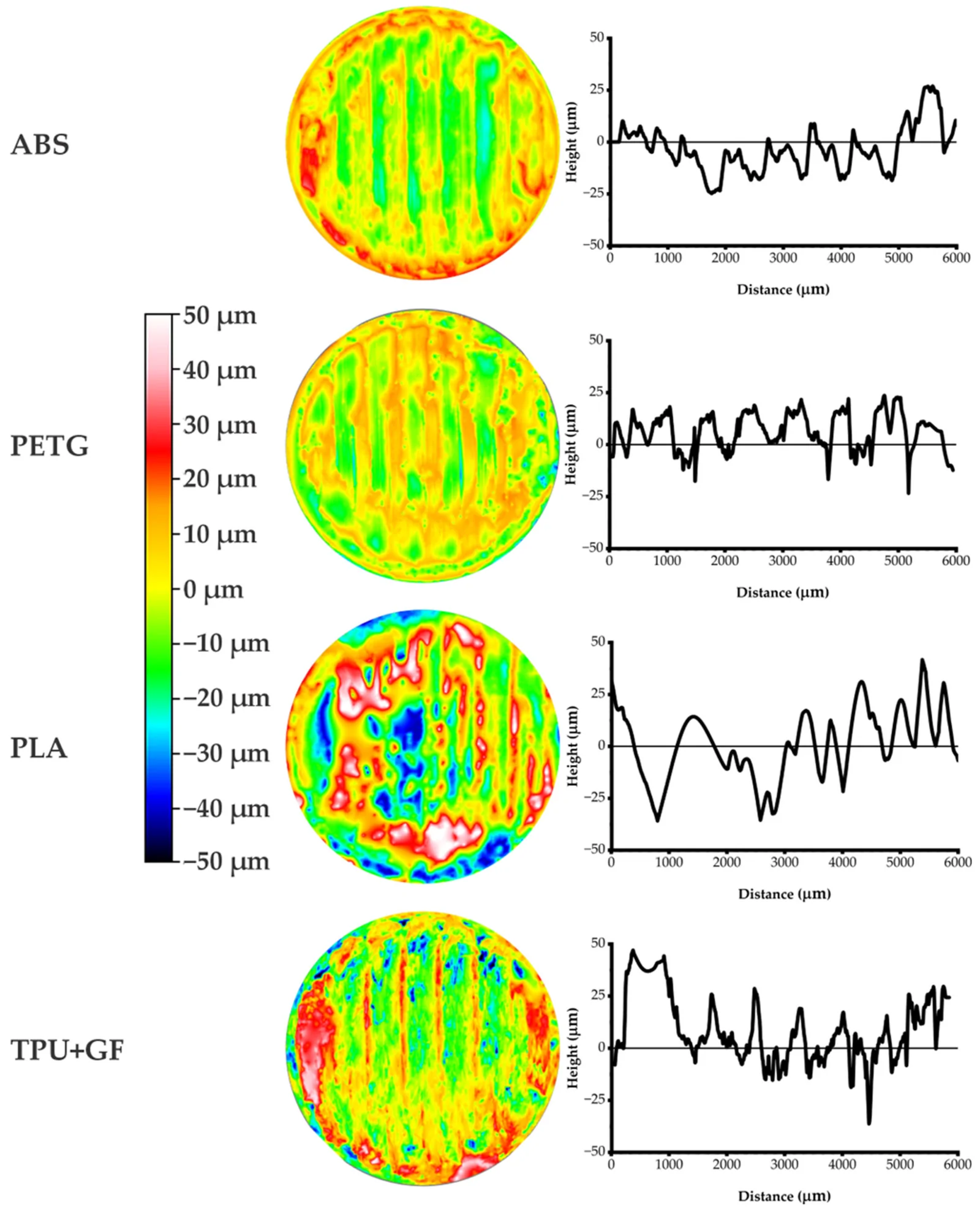
Three-dimensional surface height maps of representative specimens with profiles measured horizontally after printing.

In addition to surface roughness measurements, surface topography maps were generated for all materials investigated in this study. The resulting maps are presented in Figure 4. Alongside the areal topography images, a linear surface profile was extracted along the horizontal axis of each specimen. The corresponding profile measurements are also included in Figure 4, adjacent to the top-view representation of the specimen surface.

### 3.2. Mechanical Properties

Table 4 summarizes the hardness (H_IT_) and indentation modulus (E_IT_) of the investigated FFF-printed polymers, determined via instrumented microindentation using the Oliver–Pharr method. In addition to the mean values obtained from 15 measurements, the corresponding population standard deviation is also reported.

Table 4 shows clear differences in the mechanical response of the tested polymers. ABS, PETG, and PLA exhibit comparable hardness values, with HIT in the range of approximately 120–135 MPa, whereas TPU + GF shows substantially lower hardness of about 45–50 MPa. The indentation modulus follows a similar material-dependent trend, with PLA reaching the highest EIT value, ABS and PETG showing intermediate stiffness, and TPU + GF displaying the lowest modulus.

These differences are important for interpreting the tribological results because hardness and elastic modulus influence the real contact area, surface deformation, and load-bearing capacity during sliding. Materials with higher HIT and EIT can better resist local indentation and wear, whereas materials with lower modulus may adapt more readily to the counter surface, increasing compliance but also making them more susceptible to dimensional change under load [17,18,20,21].

Therefore, the mechanical properties provide a basis for linking material stiffness and hardness to friction and wear behavior in the water-lubricated sliding contact.

### 3.3. Friction Behavior

**Figure 5:**
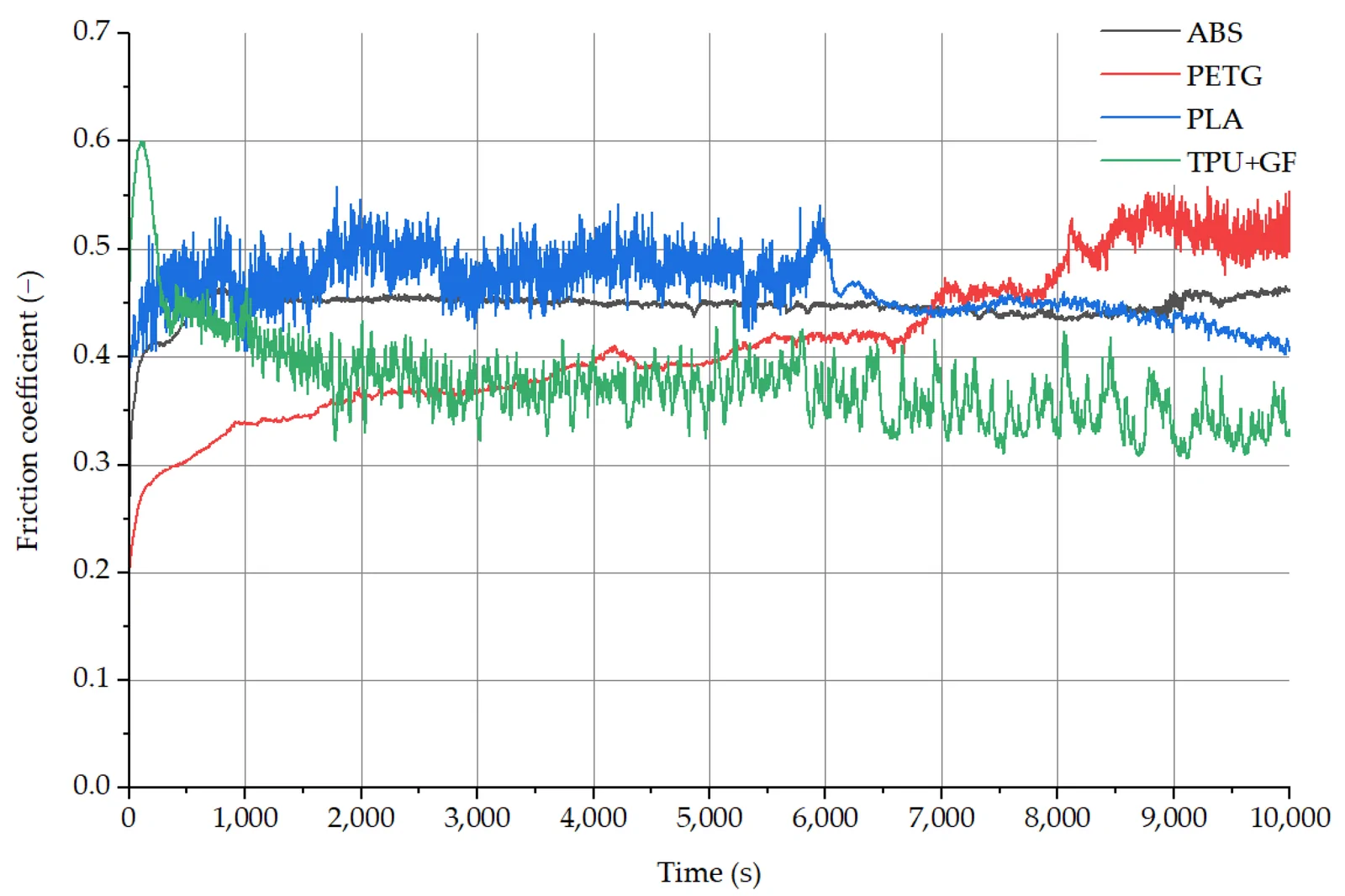
Coefficient of friction as a function of sliding time for FFF-printed polymers (ABS, PETG, PLA, and TPU + GF) under dry pin-on-disc contact conditions (P=14 N, v=1 m/s).

Representative tribological test curves obtained under pin-on-disc contact in dry conditions are presented in Figure 5. The plot shows the variation in the coefficient of friction as a function of time recorded during the tests for each of the investigated materials. The most stable coefficient of friction was observed for ABS, which reached a value of 0.45 after 900 s and exhibited a slight increase towards the end of the test. The coefficient of friction for the PETG specimen increased throughout the test, reaching the highest value of 0.55 among all investigated materials at the end of the test. In contrast, TPU + GF, unlike the other materials, reached its maximum coefficient of friction (up to 0.6) in the initial stage of the test (after 115 s). Subsequently, the value stabilized after 1800 s and remained within the range of 0.32 ÷ 0.42.

Wear was characterized by mass loss and a change in pin height. The measurement results for the pins, including the mean values and corresponding standard deviations, are summarized in Table 5. The highest wear was observed for the specimen made of PLA, which exhibited intensive wear during the test. Both the mass loss and the change in pin height were significant, amounting to 0.1277 g and 3.123 mm, respectively. A very similar mass loss was observed for materials ABS and PETG, amounting to 0.0056 g and 0.0051 g, respectively, despite considerable differences in pin height change, which reached 0.250 and 0.153 mm. The lowest wear was observed for TPU + GF, for which the mass loss was 0.0011 g, while the pin height change amounted to 0.11 mm.

### 3.4. Wear and Surface Damage

As in the pre-test analysis, the surfaces of all investigated materials were examined following the tribological tests. The results of the surface measurements are summarized in Table 6. The lowest post-test surface roughness was observed for ABS. Comparable Sa values of 2.65 µm and 2.92 µm were recorded for materials PLA and TPU + GF, respectively. The largest decrease in the Sa parameter, determined by comparing the specimen surfaces before and after the tribological tests, was found for PLA and amounted to 14.07 µm. For the remaining materials, the reductions in Sa were of a similar magnitude, ranging from 6.40 to 8.68 µm.

**Figure 6:**
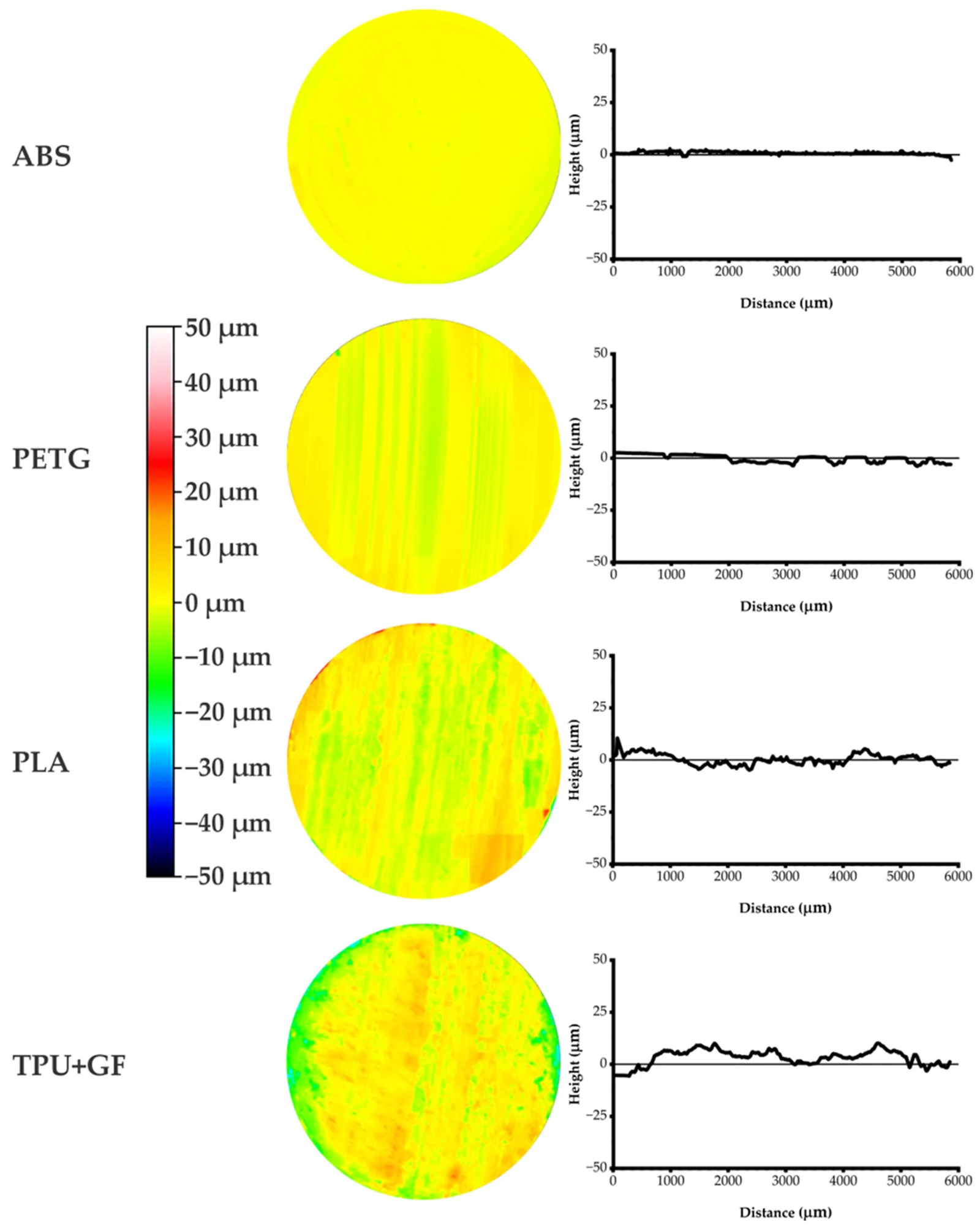
Three-dimensional surface height maps of representative specimens with profiles measured horizontally after the tests.

The surface topography results for all investigated materials are summarized in Figure 6. A considerable reduction in surface roughness was observed following the tribological tests. The lowest surface roughness and the highest degree of surface homogeneity after the tribological tests were observed for ABS. The material was characterized by a low Sa value of 8.42 µm before the test, and its coefficient of friction remained relatively stable over the majority of the testing period.

Wear marks corresponding to the movement direction of the counterface were observed on the remaining specimens. While these features were relatively uniform and limited in extent for PETG, the surfaces of PLA and TPU + GF were characterized by a more irregular topography and higher roughness. The corresponding Sa values were 1.56 µm for PETG and 2.65 µm and 2.92 µm for PETG and TPU + GF, respectively. Despite exhibiting the highest post-test surface roughness, the TPU + GF specimen demonstrated the lowest wear.

**Figure 7:**
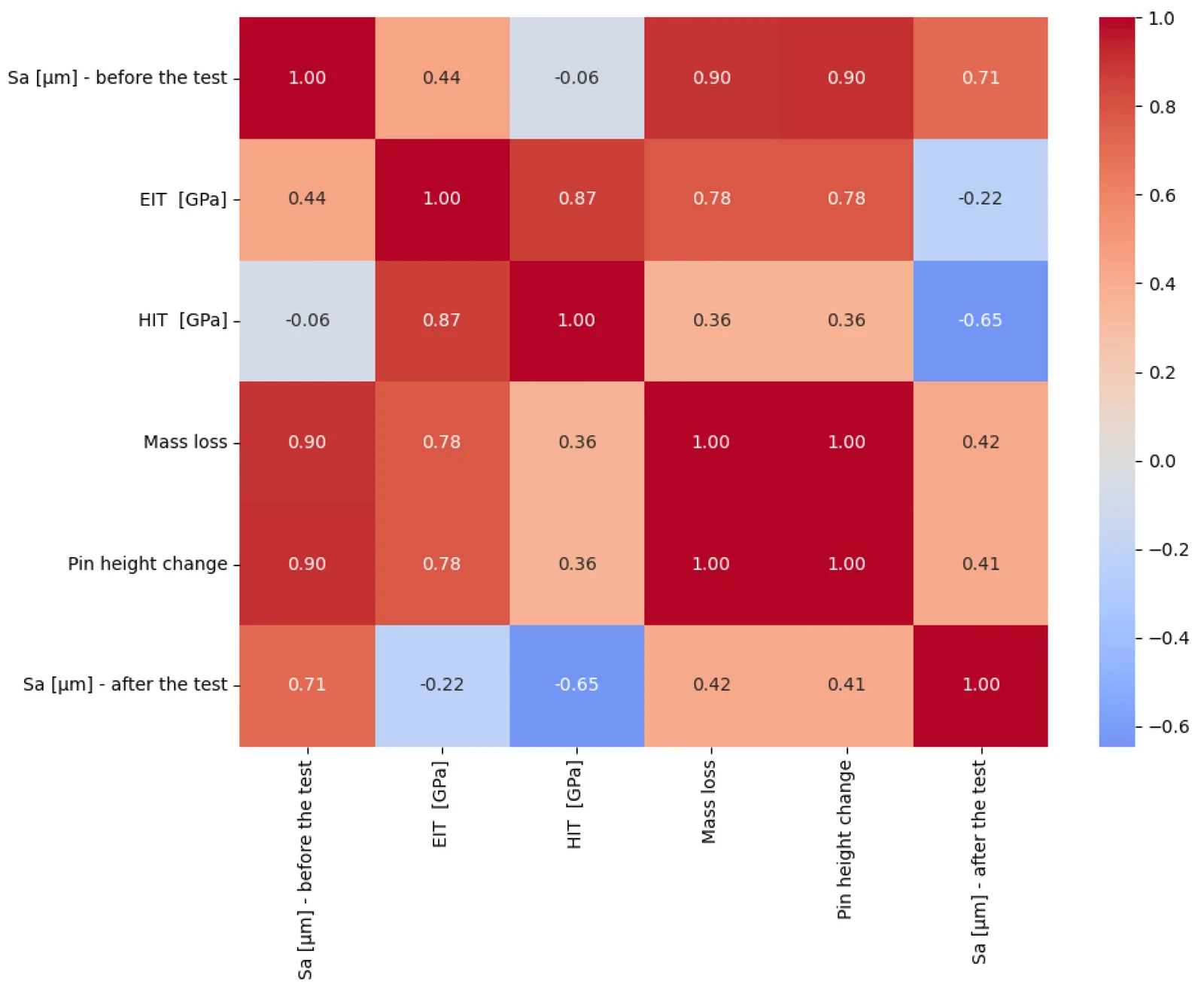
Pearson’s correlation outcomes for selected parameters.

Pearson correlation analysis presented on Figure 7 was conducted to assess the relationships among the mechanical properties, the surface roughness parameters measured before and after tribological testing, and the wear characteristics of the investigated materials, quantified by mass loss and pin height change. The correlation matrix provides valuable insight into the relationships among the analyzed variables and their contribution to the wear behavior of the investigated materials. Based on the obtained results, the following observations can be made:

- A very strong positive correlation was found between the surface roughness measured before testing and the wear indicators, namely mass loss and pin height change. These results indicate that materials characterized by greater surface irregularities are more susceptible to wear.
- The analysis revealed a weak negative correlation between hardness and surface roughness, indicating that these parameters are largely independent for the investigated materials.
- The results revealed a strong positive correlation between the elastic modulus and both mass loss and pin height change, indicating that materials characterized by higher stiffness tended to experience greater wear. Similarly, a moderate positive correlation was observed between hardness and the wear parameters, suggesting that increased hardness was generally accompanied by higher mass loss and pin height change.

## 4. Discussion

### 4.1. Surface Roughness and Wear

Surface roughness is one of the key factors affecting the tribological performance of printed polymer materials [22,23,24]. Variations in surface topography influence the contact mechanics at the sliding interface, altering the friction coefficient, wear mechanisms, and transfer film formation. Higher roughness values may increase local stress concentrations, as a limited number of surface asperities initially carry the load. This promotes abrasive wear and accelerates the removal of asperity peaks, whereas lower roughness generally provides more uniform contact conditions and improved wear resistance [25,26,27]. On the other hand, in polymer systems, wear debris may contribute to the formation of transfer films on the counterface, which can reduce both wear and friction [26].

In the investigated case, the highest Sa values were observed for PLA and TPU + GF. The increased surface roughness of these materials, relative to ABS and PETG, is consistent with the fluctuations in the coefficient of friction observed during the tests, shown on Figure 5, indicating a less stable contact at the sliding interface.

The resulting wear debris becomes trapped within the sliding interface between the specimen and the counterface, modifying the contact conditions and contributing to the fluctuations in the coefficient of friction [28] was observed for both PLA and TPU + GF; however, the wear of PLA was significantly higher than that of TPU + GF. Similar fluctuations in the coefficient of friction were observed for PETG during the final stage of the test. This behavior was related to the accumulation of wear debris in the wear track, which modified the contact conditions between the specimen and the counterface [29]. However, the wear of PLA was significantly higher, indicating a more severe abrasive wear mechanism, in contrast to TPU + GF, which exhibited the lowest wear. A notable difference was also observed in the morphology of the wear debris. In the case of PLA, the wear products took the form of flakes that, unlike those generated by PETG, did not adhere to the counterface within the wear track but instead accumulated behind the pin or were expelled from the contact area. In contrast, only powder-like wear debris was observed for TPU + GF. The observed behavior may be associated with the formation of a protective tribolayer in the case of TPU + GF, promoted by its lower hardness and the greater fragmentation of the wear debris [25,27]. In contrast, PLA, which exhibited higher hardness, generated larger and harder wear particles that acted as abrasive agents, thereby increasing wear [30].

### 4.2. Relationship Between Mechanical and Tribological Behavior

The comparison of monolithic polymers with a glass-fiber-reinforced polymer revealed a deviation from the commonly accepted relationship between mechanical properties and wear resistance [31]. Although increased hardness is generally associated with improved resistance to wear, the material that exhibited the lowest wear in the pin-on-disc tests was characterized by the lowest hardness among all investigated materials. The observed behavior was likely affected by the orientation of the reinforcing fibers, whose arrangement has a significant influence on the wear performance of this type of material [32].

**Figure 8:**
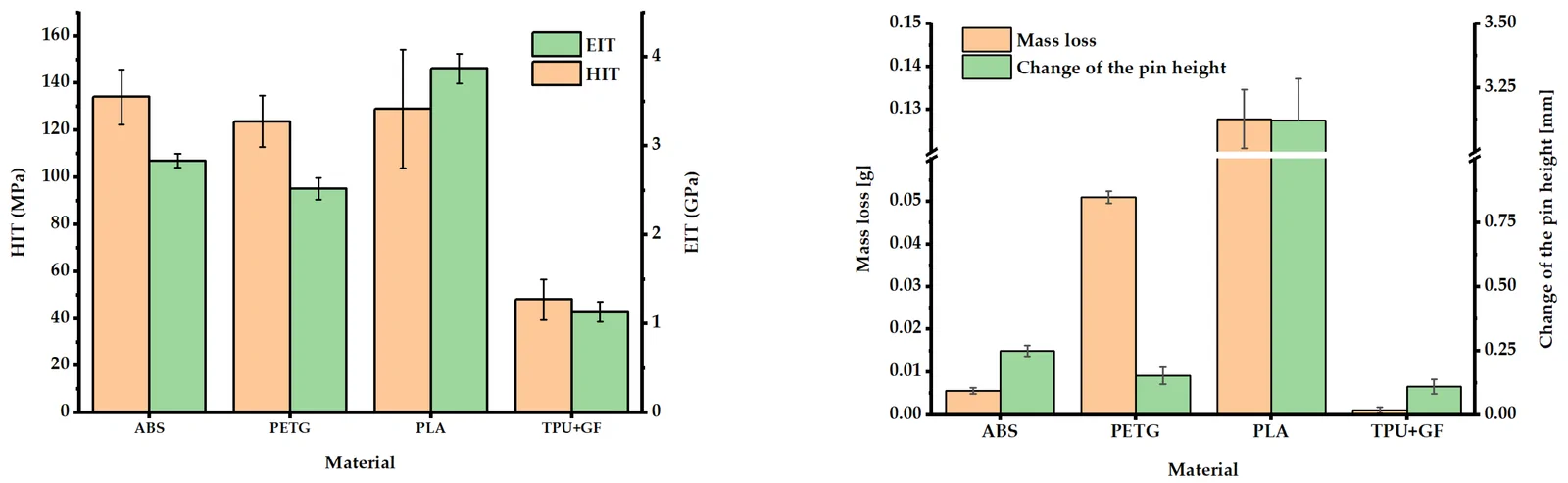
Bar charts comparing the hardness and mass loss of the investigated materials, with error bars representing the standard deviation.

A comparison of the wear results and mechanical properties, presented in Figure 8, reveals substantial wear of material PLA, which exhibited hardness values comparable to those of the other monolithic materials, while showing a noticeably higher elastic modulus. In contrast, significantly lower H_IT_ and E_IT_ values were recorded for TPU + GF, which demonstrated the lowest wear among all investigated materials. Furthermore, a comparison of ABS and PETG shows that their mass losses were nearly identical, differing by only 0.0004 g in favor of PETG, despite the hardness of ABS being more than 10 MPa higher.

## 5. Conclusions

Presented results indicate that hardness alone cannot fully explain the wear behavior of the investigated materials, suggesting that additional factors, such as wear mechanisms, wear products and tribolayer formation, may play a significant role. However, the Pearson correlation analysis identified a strong positive correlation between the elastic modulus and both mass loss and pin height change, indicating that material stiffness remained an important factor influencing wear performance.

Although the addition of reinforcing fibers to TPU + GF did not lead to an improvement in its mechanical properties, it significantly enhanced the material’s wear resistance, resulting in the best wear performance among all investigated materials.

Changes in the coefficient of friction observed during the tests indicate alterations in the contact conditions between the investigated materials and the counterface, particularly in the case of PLA, PETG, and TPU + GF. Besides the mechanical properties of the materials, their surface topography, including roughness, played an important role in this behavior. Increased surface roughness promotes uneven wear, enhances wear debris generation, and leads to less uniform operation of the sliding pair.

The unlubricated pin-on-disc tests conducted in this study represent an essential preliminary screening protocol focused on boundary friction and startup conditions. While TPU + GF demonstrated superior wear resistance under dry sliding, subsequent component-level testing under actual water-lubricated regimes will be required to evaluate its full hydrodynamic performance.

## Figures and Tables

**Table 1:** FFF printing parameters for investigated materials.

Material	Nozzle Temperature [°C]	Bed Temperature [°C]	Chamber Temp [°C]	Printing Speed [mm/s]	Infill Density [%]
PLA	220	60	40	50–100	100
PETG	250	80	50	40–80	100
ABS	260	100	55	40–70	100
TPU + GF	240	70	45	20–40	100

**Table 2:** Tribological test parameters used in the pin-on-disc experiments.

Load applied to pin	14 N
Nominal contact pressure	0.5 MPa
Friction radius	51.5 mm
Disc rotational speed	186 rpm
Linear sliding velocity	1 m/s
Friction type	dry
Pin diameter	6 mm
Pin height	12 mm

**Table 3:** Roughness of printed samples measured in accordance with the ISO 25178 standard [18].

	ABS	PETG	PLA	TPU + GF		
Sp	33.75	30.80	55.30	50.58	µm	Maximum peak height
Sv	26.29	64.59	41.88	67.92	µm	Maximum pit height
Sz	60.04	95.39	97.18	118.5	µm	Maximum height
Sa	8.42	7.96	16.72	11.60	µm	Arithmetic mean height
Sq	10.38	9.69	20.67	14.94	µm	Root mean square height
Ssk	0.153	−0.301	0.273	0.172		Skewness

**Table 4:** Mechanical properties of the investigated materials—mean values and standard deviations (σ).

Material	E_IT_ [GPa]	σ	H_IT_ [MPa]	σ
ABS	2.52	0.07	134.18	11.65
PETG	2.24	0.11	123.78	10.84
PLA	3.44	0.15	129.01	25.14
TPU + GF	1.01	0.10	48.05	8.56

**Table 5:** Mass loss and pin height change in the investigated materials after tribological testing (mean values and corresponding standard deviations).

Material	Mass Loss [g]	σ	Pin Height Change [mm]	σ
ABS	0.0056	0.0007	0.250	0.010
PETG	0.0051	0.0014	0.153	0.033
PLA	0.1277	0.0196	3.123	0.043
TPU + GF	0.0011	0.0007	0.110	0.028

**Table 6:** Roughness of tested samples measured in accordance with the ISO 25178 standard [18].

	ABS	PETG	PLA	TPU + GF		
Sp	3.39	5.56	35.96	17.29	µm	Maximum peak height
Sv	3.67	14.51	37.02	26.49	µm	Maximum pit height
Sz	7.06	20.07	72.97	43.78	µm	Maximum height
Sa	0.489	1.56	2.65	2.92	µm	Arithmetic mean height
Sq	0.66	1.84	3.59	3.72	µm	Root mean square height
Ssk	−0.3748	−0.1481	0.9939	−0.0726		Skewness

## Data Availability

The original contributions presented in this study are included in the article. Further inquiries can be directed to the corresponding author.
